# Radiographic involvement of cervical facet joints in ankylosing spondylitis: a longitudinal analysis in correlation with vertebral body lesions

**DOI:** 10.1186/s41927-023-00334-x

**Published:** 2023-06-07

**Authors:** Tae-Han Lee, Seunghun Lee, Bon San Koo, Kyung Bin Joo, Tae-Hwan Kim

**Affiliations:** 1 Department of Rheumatology, Keimyung University Dongsan Hospital, Daegu, South Korea; 2 Department of Radiology, Hanyang University Hospital, Seoul, South Korea; 3 Division of Rheumatology, Department of Internal Medicine, Inje University Seoul Paik Hospital, Seoul, South Korea; 4 Department of Rheumatology, Hanyang University Hospital for Rheumatic Diseases, 222-1, Wangsimni-ro, Seongdong-gu, Seoul, 04763 South Korea

**Keywords:** Ankylosing spondylitis, Facet joint, Radiography, Syndesmophyte

## Abstract

**Background:**

The inability to assess structural changes in facet joints is a limitation of established radiographic scoring systems for ankylosing spondylitis (AS). We compared radiographic evidence of ankylosis in cervical facet joints and cervical vertebral bodies in patients with AS.

**Methods:**

We analysed longitudinal data collected from 1106 AS patients and assessed 4984 spinal radiographs obtained up to 16 years of follow-up. Comparisons between cervical facet joints and cervical vertebral bodies focused on the presence of ankylosis, which was defined by at least one facet joint exhibiting complete ankylosis (according to the method of de Vlam) or at least one vertebral body with a bridging syndesmophyte (according to the modified Stoke Ankylosing Spondylitis Spinal Score [mSASSS]). Ankylosis was assessed over time using spinal radiographs collected during follow-up periods stratified in 4-year increments.

**Results:**

Patients with cervical facet joint ankylosis had higher cervical mSASSS, sacroiliitis grades, and inflammatory markers, with more prevalent hip involvement and uveitis. Overall, the numbers of spinal radiographs indicating ankylosis were comparable between cervical facet joints (17.8%) and cervical vertebral bodies (16.8%), and they usually presented together (13.5%). We observed similar proportions of radiographs with ankylosis only in cervical facet joints (4.3%) and cervical vertebral bodies (3.3%). As damage progressed, configurations with both cervical facet joint ankylosis and bridging syndesmophytes became more predominant with longer follow-up times, while configurations with cervical facet joint ankylosis only or bridging syndesmophytes only were less frequently observed.

**Conclusions:**

Evidence of cervical facet joint ankylosis appears as often as bridging syndesmophytes on routine AS spinal radiographs. Presence of cervical facet joint ankylosis should be considered because it may have a higher disease burden.

**Supplementary Information:**

The online version contains supplementary material available at 10.1186/s41927-023-00334-x.

## Background

Inflammatory manifestations in the axial skeleton followed by new bone formation, presenting as syndesmophytes and associated bridging, are prominent features of ankylosing spondylitis (AS) [1]. To quantify these structural changes, several scoring methods have been developed, including the modified Stoke Ankylosing Spondylitis Spinal Score (mSASSS), which is the most validated and widely used [2]. These scoring systems focus on evaluating lesions in the vertebrae; thus, assessment of the presence of syndesmophytes is a key outcome measure in clinical studies [2, 3].

Facet joints, which are crucial anatomic structures consisting of posterior arches of the spine, can also be affected in AS, demonstrating typical osteoproliferative changes such as ankylosis [4, 5]. Because facet joints, in conjunction with anterior spinal structures, play important roles in controlling the motions of the spine during bearing and transmission of mechanical loads [6], impaired mechanical function due to ankylosis of this joint can cause further deterioration in spinal mobility in patients with AS. Growing clinical evidence suggests the functional relevance of facet joint involvement in AS [7–11]. However, facet joints tend to be overlooked in AS compared with structural changes in the vertebral bodies and are not usually included in established scoring systems like the mSASSS [2]. The curved configuration of the facet joint or overlying ribs and lung structures can limit radiographic assessment of this joint, particularly at the levels of the lumbar and thoracic spines [6, 12, 13]. To date, only one radiographic scoring method has been proposed for assessment of facet joint involvement in AS. According to de Vlam et al. [14], radiographic abnormalities of facet joints in the cervical spine can be scored with good reproducibility to identify ankylosis.

Some studies have compared radiographic involvement of cervical facet joints and cervical vertebral bodies in patients with AS. Earlier studies reported that 26%–54% of patients with longstanding AS had cervical facet joint ankylosis, which was a greater frequency than those with (bridging) syndesmophytes [4, 14, 15]. In contrast, a 4-year follow-up study by Maas et al. showed that radiographic-detectable damage of cervical vertebral bodies was nearly twice as prevalent as that of cervical facet joints [16]. However, long-term outcomes for facet joint damage, along with the association between facet joint ankylosis and (bridging) syndesmophytes, have yet to be reported. This study aimed to compare the status of radiographic involvement of cervical facet joints over time with those of cervical vertebral bodies in patients with AS.

## Methods

We retrospectively reviewed the records of consecutive AS patients who fulfilled the modified New York criteria [17] at a single hospital between January 2001 and December 2018. Longitudinal data were collected from patients for whom the results of radiographic assessment of cervical facet joints and cervical vertebral bodies were available. This study was approved by the institutional review board of our university hospital (HYUH 2021-10-004), and the need for patient consent was waived because of the retrospective nature of the study.

## Clinical assessment

The following information on demographics and clinical characteristics was obtained: age, sex, symptom duration, smoking status, human leucocyte antigen (HLA)-B27 status, serum erythrocyte sedimentation rate (ESR), C-reactive protein (CRP), disease activity as determined by the Bath Ankylosing Spondylitis Disease Activity Index (BASDAI), and a dichotomous measure of exposure to tumour necrosis factor (TNF) inhibitors during follow-ups.

## Radiographic assessment

Two radiologists (SL and KBJ) and a trained rheumatologist (T-HL) assessed individual skeletal regions on radiographs. Assessment was conducted in the chronological order in which the radiographs were obtained, and the readers were blinded to patients’ clinical data. Since evaluation of vertebral bodies in the cervical and lumbar spines had been completed in our preceding investigation [18], radiographic assessments of cervical vertebral bodies and cervical facet joints were performed at different time points. Cervical vertebral bodies were independently scored by two readers (SL and KBJ) according to the mSASSS [19]. Intra-observer and inter-observer reliabilities were both excellent, with intra-class correlation coefficients (ICCs) of 0.98 (95% confidence interval [CI], 0.98–0.98) and 0.95 (95% CI, 0.94–0.95), respectively [18].

**Fig. 1:**
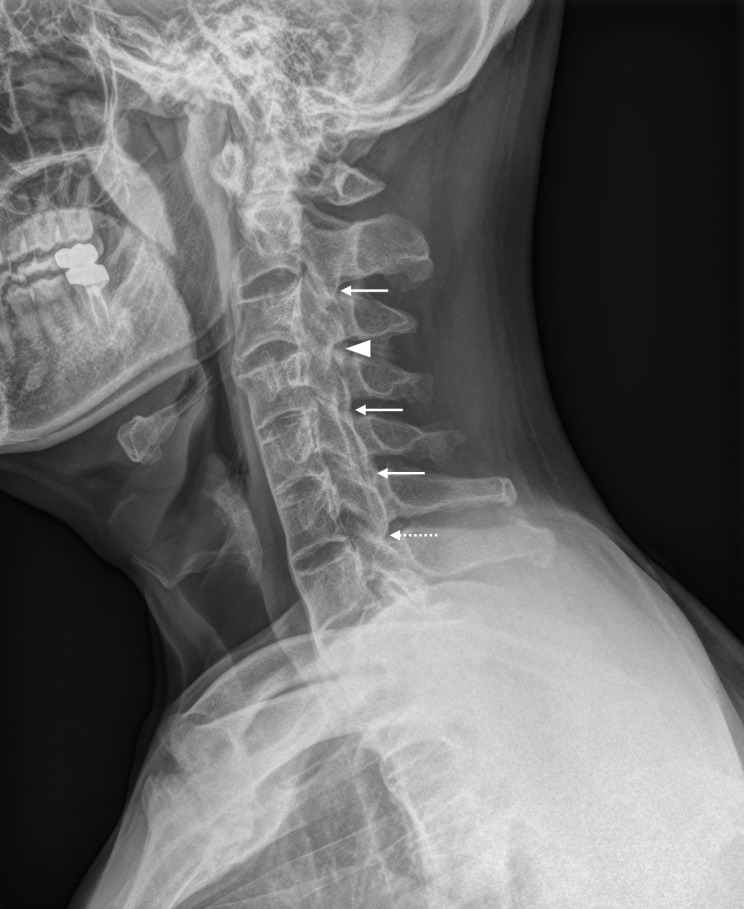
A lateral radiograph of the cervical spine obtained from a 39-year-old ankylosing spondylitis patient with a disease duration of 18 years. At C3–C4 level, cervical facet joint with complete ankylosis is seen (arrowhead, a score of 3 according to de Vlam et al. [14]). Partial ankylosis and joint space narrowing is seen at C2–C3, C4–C5, and C5–C6 (white arrows, a score of 2) and C6–C7 level (dashed arrow, a score of 1), respectively. On the anterior side of the spine, bridging syndesmophtyes are seen

For assessment of cervical facet joints, we used the scoring method proposed by de Vlam et al. [14]. Based on the lateral views of spinal radiographs, facet joints on one side in individual inter-vertebral levels from C2–C3 to C6–C7 were scored as follows: 0 = normal, 1 = joint space narrowing or erosion, 2 = partial blurring or ankylosis, and 3 = complete blurring or ankylosis (Fig. 1). The total score was calculated as the sum of the scores of five individual facet joints (range, 0–15). First, as a preliminary analysis, two readers (T-HL and SL) independently scored cervical facet joints using a subset of data (271 spinal radiographs). Inter-observer reliability for the total cervical facet joint score was excellent, with an ICC of 0.92 (95% CI, 0.90–0.93). Subsequently, one reader (T-HL) scored all included cervical facet joints, and these values were used for analysis. Because we were interested in comparing radiographically observed involvement status between cervical facet joints and cervical vertebral bodies at given levels of damage, the appearance of at least one cervical facet joint with complete ankylosis (score of 3 according to de Vlam et al.) and at least one cervical vertebral body with a bridging syndesmophyte (score of 3 according to mSASSS) were accepted as indicative of the presence of ankylosis in each structure [16].

Grading of radiographic sacroiliitis and hip joint damage was performed by one reader (T-HL) according to the modified New York criteria [17] and the Bath Ankylosing Spondylitis Radiology Hip Index (BASRI-hip; grades 0–4), respectively [20]. Hip involvement was defined as the presence of definite narrowing (grade ≥ 2) in at least one side of both hips [21].

## Statistical analyses

Results were expressed as numbers (%), means ± standard deviations (SDs) or median (interquartile range [IQR]) for categorical, normally distributed and non-normally distributed data, respectively. Descriptive statistics were used to analyse the characteristics of the study population. When comparing demographic and clinical outcomes between patients with and without involvement of the cervical facet joints, continuous variables were analysed using the independent-samples *t* test or Mann–Whitney *U* test. Categorical variables were compared between the groups using the chi-square test or Fisher’s exact test, as appropriate. With the patient as the unit of analysis, correlations between cervical facet joint scores and disease variables were assessed using Pearson’s correlation coefficient.

Since the durations of follow-up and the intervals between consecutive spinal radiographs varied by patient, the entire follow-up period was stratified into 4-year increments to account for radiographic outcomes over time. Thus, we used spinal radiographs collected within the stratified follow-up period as the unit of analysis when comparing radiographic evidence of ankylosis between cervical facet joints and cervical vertebral bodies. Patients could contribute several spinal radiographs within a given follow-up period. Considering differences in the ranges of vertebral levels evaluated by the method of de Vlam (C2–C3 to C6–C7) and cervical mSASSS (C2–C3 to C7–T1), the C7–T1 level was not included when assessing radiographic involvement of each structure, as defined above. Therefore, a comparison with respect to the presence or absence of ankylosis between facet joints and vertebral bodies was performed at the C2–C3 to C6–C7 vertebral levels. The distribution of ankylosed facet joints and bridging syndesmophytes was also evaluated per individual vertebral level from C2–C3 to C6–C7. R statistical language version 4.0.4 was used for the analyses. *P* < 0.05 was considered statistically significant.

## Results

**Fig. 2:**
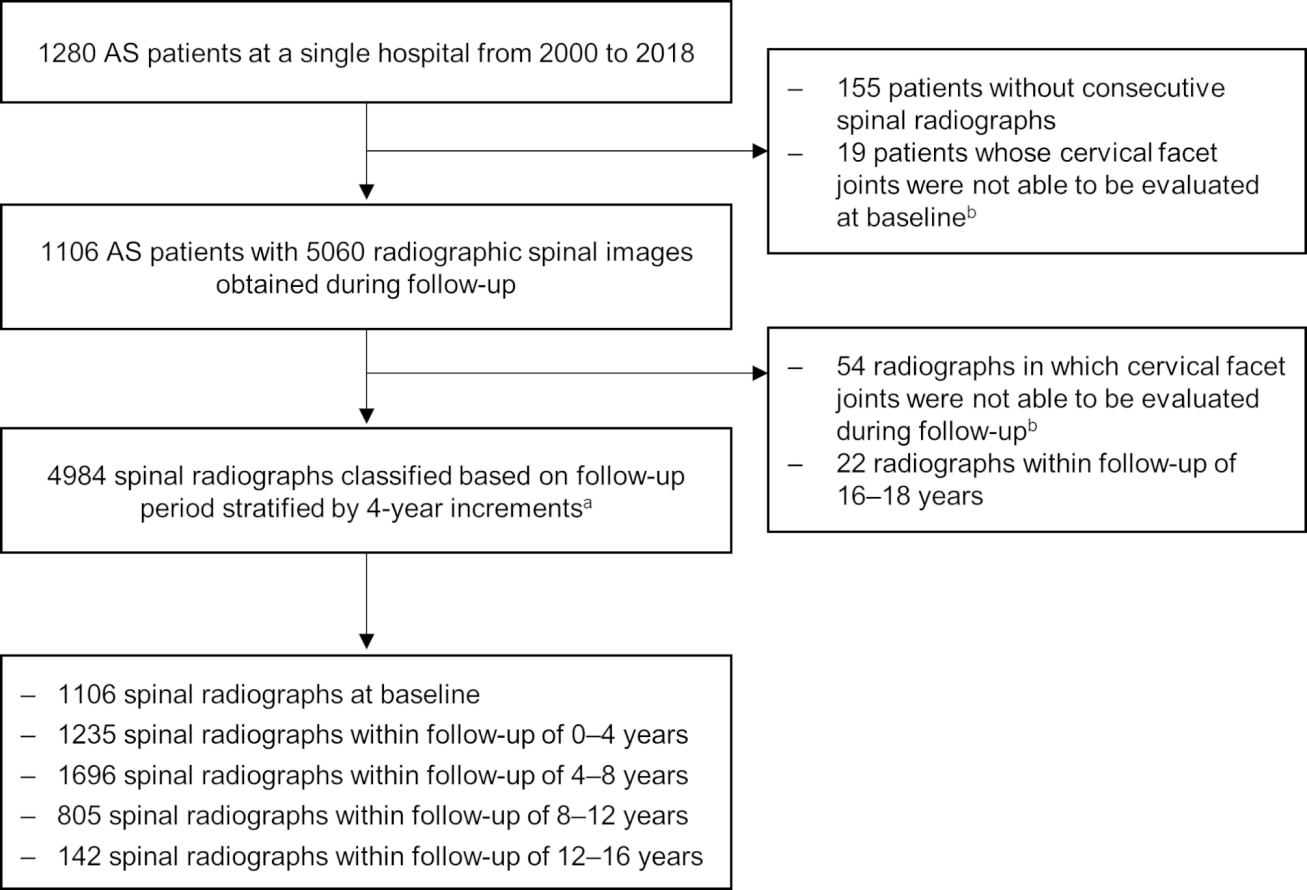
Flowchart of included ankylosing spondylitis patients with spinal radiographic data. Several spinal radiographs within the given follow-up period were included per patient. (^a^Spinal radiographs were collected during any time point within the corresponding follow-up period. ^b^Cervical facet joints that were not visible on lateral radiographs or could not be evaluated due to spinal surgery)

Among the 1280 patients screened, 1106 were included in this study after excluding those without at least two sets of spinal radiographs and those with cervical facet joints that proved difficult to assess: not visible on lateral radiographs or could not be evaluated due to spinal surgery. During follow-up, a total of 4984 spinal radiographs were obtained from the included patients, with a mean ± SD follow-up duration of 8.3 ± 2.8 years per patient (median 8.1 years, range 1.5–15.9 years). Spinal radiographs were then classified based on duration of follow-up at time of collection (Fig. 2): baseline (*n* = 1106), 0 to 4 years (*n* = 1235), 4 to 8 years (*n* = 1696), 8 to 12 years (*n* = 805), and 12 to 16 years (*n* = 142).

## Characteristics of patients with or without cervical facet joint ankylosis

Demographic, clinical, and radiographic characteristics are shown in Table 1. At baseline, 1106 patients had a mean ± SD age of 31.4 ± 9.4 years and median (IQR) symptom duration of 7 (3–13) years. At baseline, 131 (11.8%) and 121 (10.9%) patients had ankylosis of at least one cervical facet joint and at least one bridging syndesmophyte, respectively. As radiographically observable damage progressed over time, patients with ankylosis of at least one cervical facet joint or at least one bridging syndesmophyte during any follow-up period composed 230 (20.8%) and 220 (19.9%) of the study population, respectively.

**Table 1:** Characteristics of the study population, both overall and stratified by the presence or absence of cervical facet joint ankylosis during any period of follow-up

	No. with data	Total patients (*n* = 1106)	≥ 1 cervical facet joint ankylosis		
			Presence (*n* = 230)	Absence (*n* = 876)	*P*-value
Baseline parameters					
Age, mean ± SD years	1106	31.4 ± 9.4	34.8 ± 8.2	30.5 ± 9.5	< 0.001
Male sex, *n* (%)	1106	976 (88.2%)	220 (95.7%)	756 (86.3%)	< 0.001
Symptom duration, years	892	7.0 (3.0–13.0)	11.5 (6.0–17.0)	6.0 (2.0–12.0)	< 0.001
HLA-B27 positivity, *n* (%)	1101	1062 (96.5%)	225 (97.8%)	837 (96.1%)	0.288
ESR, mm/h	865	22.0 (7.0–46.0)	40.0 (19.0–60.0)	18.0 (5.0–41.5)	< 0.001
CRP, mg/dL	705	1.2 (0.8–2.7)	1.9 (1.3–3.3)	1.0 (0.8–2.3)	< 0.001
BASDAI (0–10), mean ± SD	263	5.1 ± 2.7	5.5 ± 2.6	5.0 ± 2.7	0.162
Sacroiliitis grade (0–4)^a^, mean ± SD	1052	2.8 ± 0.9	3.5 ± 0.6	2.6 ± 0.9	< 0.001
Hip involvement^b^, *n* (%)	1084	110 (10.1%)	41 (18.6%)	26 (3.1%)	< 0.001
Cervical facet joint score (0–15)	1106	0.0 (0.0–0.0)	6.0 (2.0–13.0)	0.0 (0.0–0.0)	< 0.001
Cervical mSASSS (0–36)	1106	6.0 (4.0–8.7)	14.4 (7.6–28.0)	5.5 (3.9–7.1)	< 0.001
Total mSASSS (0–72)	1106	7.5 (5.0–15.8)	27.0 (11.8–51.8)	6.7 (5.0–10.0)	< 0.001
≥ 1 bridging syndesmophyte at baseline, *n* (%)	1106	167 (15.1%)	110 (47.8%)	11 (1.3%)	< 0.001
Longitudinal parameters					
Duration of follow-up, mean ± SD years	1106	8.3 ± 2.8	9.0 ± 2.9	8.1 ± 2.8	< 0.001
No. of spinal radiographs, mean ± SD	1106	4.5 ± 1.2	4.8 ± 1.3	4.4 ± 1.2	< 0.001
Smoking ever, *n* (%)	1052	639 (60.7%)	159 (72.3%)	480 (57.7%)	< 0.001
Uveitis ever, *n* (%)	930	352 (37.8%)	98 (47.1%)	254 (35.2%)	0.002
Peripheral arthritis ever, *n* (%)	920	391 (42.5%)	68 (33.0%)	323 (45.2%)	0.002
Use of TNF inhibitors ever, *n* (%)	1106	580 (52.4%)	153 (66.5%)	427 (48.7%)	< 0.001
≥ 1 bridging syndesmophyte during any period of follow-up, *n* (%)	1106	299 (27.0%)	186 (80.9%)	34 (3.9%)	< 0.001

Values are presented as median (interquartile range) for continuous variables unless indicated otherwise and *n* (%) for categorical variables

^a^Calculated by averaging the grades of the right and left sacroiliac joints according to the modified New York criteria

^b^Defined as a BASRI-hip score of at least grade 2

BASDAI, Bath Ankylosing Spondylitis Disease Activity Index; BASRI-hip, Bath Ankylosing Spondylitis Radiology Hip Index; CRP, C-reactive protein; ESR, erythrocyte sedimentation rate; HLA, human leucocyte antigen; mSASSS, modified Stoke Ankylosing Spondylitis Spine Score; SD, standard deviation; TNF, tumour necrosis factor

Comparisons between patients with and without ankylosis in at least one cervical facet joint were performed based on observations during the entire follow-up period. Patients with facet joint involvement were more frequently male and older and had longer symptom durations at baseline. Overall, patients with radiographic evidence of ankylosis in at least one cervical facet joint manifested a high disease burden, showing greater baseline damage of cervical vertebral bodies, sacroiliac joints, and hip joints. Additionally, a history of uveitis was more prevalent, and use of TNF inhibitors was more common, among patients with cervical facet joint ankylosis during all follow-up periods, whereas peripheral arthritis was less prevalent (Table 1).

## Relationship between cervical facet joint ankylosis and bridging syndesmophytes

Correlation analysis using baseline measures revealed a significant correlation between cervical facet joint score and cervical mSASSS (r = 0.717, *P* < 0.001). The cervical facet joint score was positively correlated with age, symptom duration, ESR, sacroiliitis grade, and BASRI-hip grade (Supplementary Table 1).

**Fig. 3:**
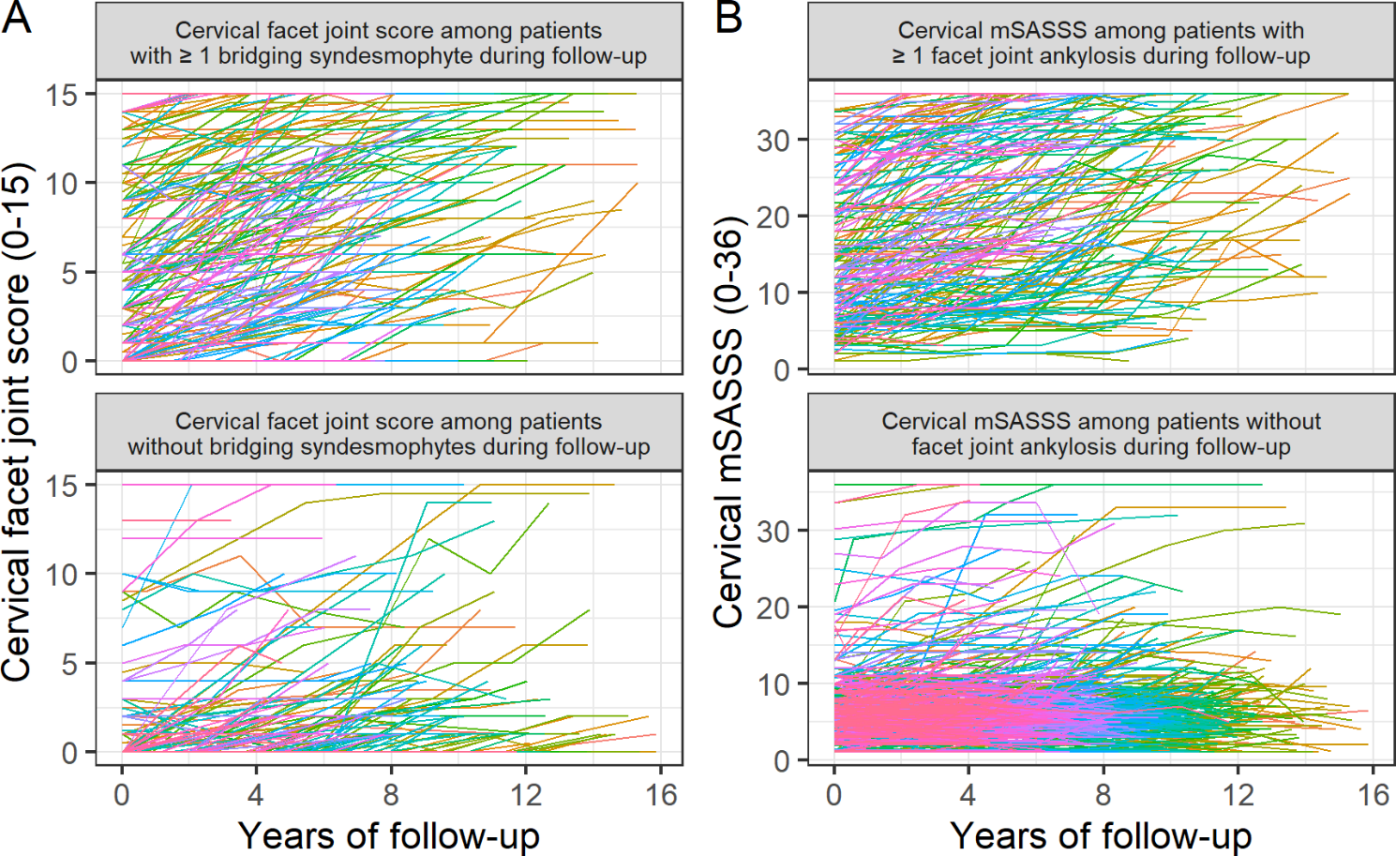
Radiographic damage scores over time at the patient level. Each line represents the progression in cervical mSASSS or cervical facet joint score for each respective patient. (A) Trends in cervical facet joint score over time as assessed among patients with or without at least one bridging syndesmophyte that occurred during any follow-up period. (B) Trends in cervical mSASSS over time as assessed among patients with or without at least one facet joint ankylosis that occurred during any follow-up period mSASSS, modified Stoke Ankylosing Spondylitis Spine Score

In Fig. 3A, individual cervical facet joint scores over time for patients with bridging syndesmophytes during any follow-up period are compared with those of patients without evidence of bridging syndesmophytes. The image shows that patients with at least one bridging syndesmophyte tended to experience greater radiographic changes in the cervical facet joints. A comparable pattern was observed for cervical mSASSS with or without ankylosis involving at least one cervical facet joint during any follow-up period (Fig. 3B). These findings suggest a close relationship between cervical facet joints and cervical vertebral bodies as radiographic evidence of ankylosis.

## Course of radiographic involvement of cervical facet joints and cervical vertebral bodies

Table 2 shows the course of radiographic involvement, which was assessed using individual spinal radiographs derived from the total patient population. Throughout the follow-up period stratified by 4-year increments, those with ankylosis in at least one cervical facet joint and at least one bridging syndesmophyte increased at a similar frequency, from 11.8% to 31.7% for cervical facet joints and 10.9% to 31.7% for cervical vertebral bodies. With respect to the extent of involvement, the numbers of vertebral levels affected by facet joint ankylosis was similar to those affected by bridging syndesmophytes.

**Table 2:** Radiographic outcomes of cervical facet joints and cervical vertebral bodies over time as assessed using individual spinal radiographs

	Total spinal radiographs over follow-up (*n* = 4984)				
	Baseline (*n* = 1106)	Y0–Y4 (*n* = 1235)	Y4–Y8 (*n* = 1696)	Y8–Y12 (*n* = 805)	Y12–Y16 (*n* = 142)
Radiographic damage of cervical facet joints					
Cervical facet joint score (0–15), mean ± SD	1.5 ± 3.9	1.8 ± 4.2	2.3 ± 4.6	3.1 ± 5.2	4.1 ± 5.7
Median (IQR)	0.0 (0.0–0.0)	0.0 (0.0–0.0)	0.0 (0.0–1.0)	0.0 (0.0–4.0)	0.0 (0.0–8.5)
≥ 1 facet joint ankylosis, *n* (%)	130 (11.8%)	186 (15.1%)	325 (19.2%)	199 (24.7%)	45 (31.7%)
Ankylosis of all facet joints, *n* (%)	44 (4.0%)	64 (5.2%)	104 (6.1%)	60 (7.5%)	12 (8.5%)
No. of affected vertebral levels (0–5)^a^, mean ± SD	0.4 ± 1.2	0.5 ± 1.3	0.6 ± 1.4	0.8 ± 1.6	1.1 ± 1.8
Median (IQR)	0.0 (0.0–0.0)	0.0 (0.0–0.0)	0.0 (0.0–0.0)	0.0 (0.0–0.0)	0.0 (0.0–2.0)
Radiographic damage of cervical vertebral bodies					
Cervical mSASSS (0–36), mean ± SD	8.4 ± 7.8	9.3 ± 8.6	10.2 ± 9.6	11.4 ± 10.6	13.7 ± 11.4
Median (IQR)	6.0 (4.0–8.7)	6.5 (4.4–9.6)	6.5 (4.4–10.8)	7.0 (4.4–14.0)	9.0 (5.3–21.7)
≥ 1 bridging syndesmophyte, *n* (%)	121 (10.9%)	175 (14.2%)	306 (18.0%)	190 (23.6%)	45 (31.7%)
Bridges of all vertebral bodies, *n* (%)	37 (3.3%)	48 (3.9%)	103 (6.1%)	65 (8.1%)	16 (11.3%)
No. of affected vertebral levels (0–5)^b^, mean ± SD	0.3 ± 1.0	0.4 ± 1.2	0.6 ± 1.4	0.8 ± 1.6	1.1 ± 1.8
Median (IQR)	0.0 (0.0–0.0)	0.0 (0.0–0.0)	0.0 (0.0–0.0)	0.0 (0.0–0.0)	0.0 (0.0–1.0)

Values are presented as mean ± SD or median (IQR) for continuous variables and *n* (%) for categorical variables

^a^Number of vertebral levels with the presence of complete facet joint ankylosis

^b^Number of vertebral levels with the presence of bridging syndesmophytes

IQR, interquartile range; mSASSS, modified Stoke Ankylosing Spondylitis Spine Score; SD, standard deviation; Y, follow-up year

## Configuration of cervical facet joint ankylosis and bridging syndesmophytes over time

**Fig. 4:**
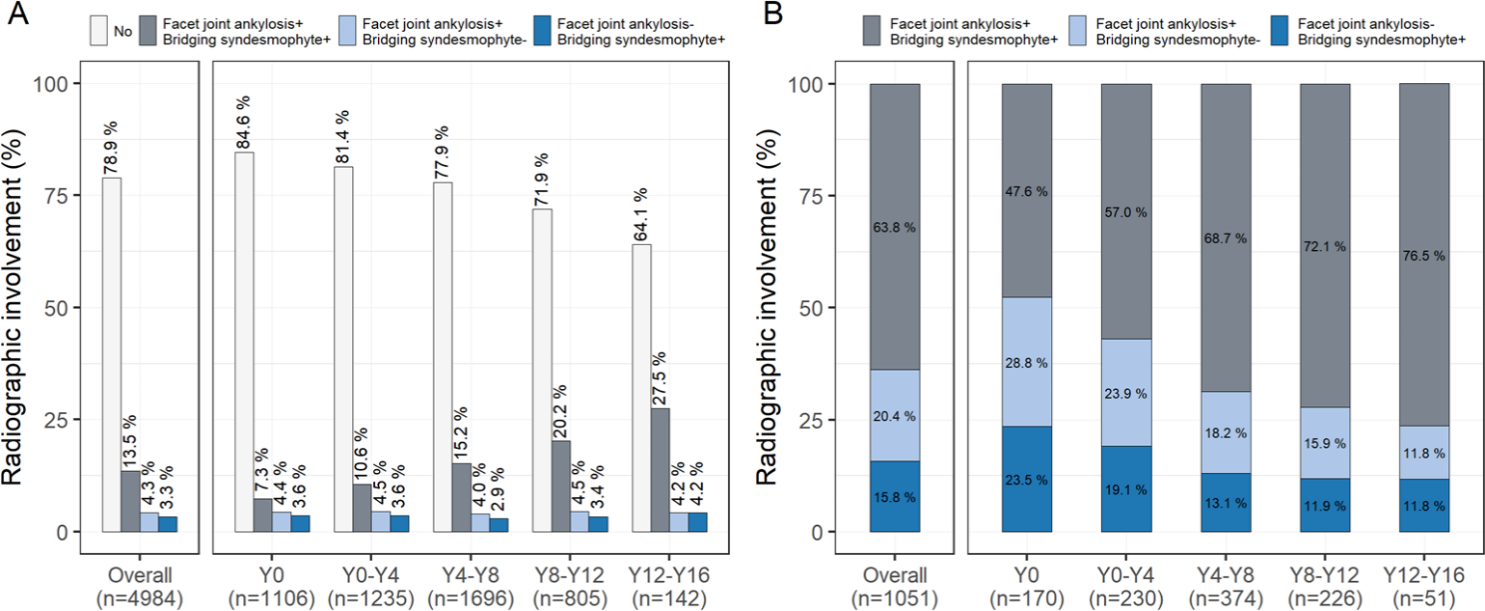
Configuration of cervical facet joint ankylosis and bridging syndesmophytes as assessed during the entire or stratified follow-up periods based on (A) total spinal radiographs and (B) spinal radiographs in the presence of either cervical facet joint ankylosis or bridging syndesmophytes. Each bar shows the frequency of the presence and/or absence of at least one ankylosis in facet joints or vertebral bodies at a given time period for the C2–C3 to C6–C7 vertebral level Y, follow-up year

Of 4984 spinal radiographs from the entire follow-up period, 17.8% presented with ankylosis in at least one cervical facet joint, and 16.8% presented with at least one bridging syndesmophyte; 13.5% exhibited ankylosis in both cervical facet joints and cervical vertebral bodies. However, radiographs indicating cervical facet joint ankylosis but no bridging syndesmophytes and those with bridging syndesmophytes but no ankylosed cervical facet joints accounted for 4.3% and 3.3% of total spinal radiographs, respectively (Fig. 4A).

In terms of the configurations identified among those with ankylosis in either the cervical facet joints or cervical vertebral bodies, radiographs with facet joint ankylosis only or those with bridging syndesmophytes only, although fewer in number, were more frequent in the early follow-up phase. However, the frequency of such configurations gradually decreased in proportion to increased radiographs presenting ankylosis of both structures (Fig. 4B). These findings imply that if either cervical facet joint ankylosis or bridging syndesmophytes are present, they usually appear together and are more likely to both be observed over time, although not always concurrently, during the disease course.

## Cervical facet joint ankylosis and bridging syndesmophytes per vertebral level

**Fig. 5:**
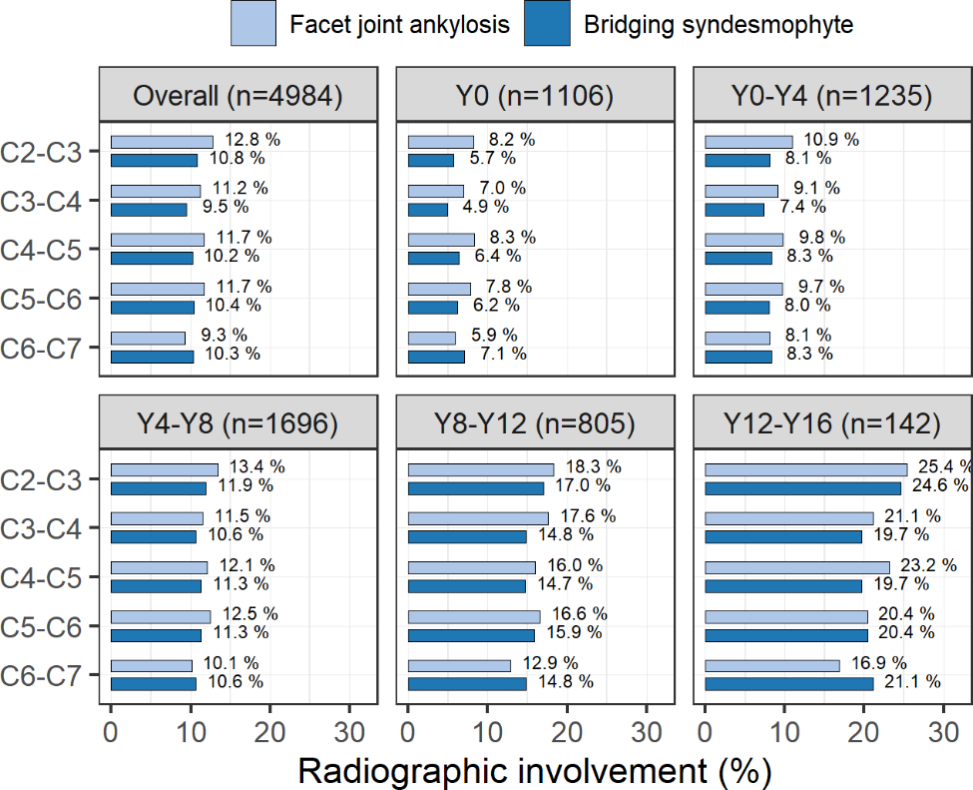
Distribution of cervical facet joint ankylosis and bridging syndesmophytes per individual vertebral level as assessed during the entire or stratified follow-up periods. Each bar shows the frequency of facet joint ankylosis or bridging syndesmophytes at a single vertebral level for a given time period Y, follow-up year

The distribution of ankylosis per individual vertebral level indicated that cervical facet joints were more frequently affected at the C2–C3 level and less so at the C6–C7 level, whereas bridging syndesmophytes were distributed more evenly throughout the cervical spine, with a slightly reduced frequency at the C3–C4 level (Fig. 5).

## Discussion

In this study, we conducted a longitudinal investigation about the radiographic involvement of cervical facet joints, which was defined by the presence of complete ankylosis, compared with bridging syndesmophytes in AS patients. During follow-up, radiographic evidence of cervical spinal ankylosis was comparable between facet joints and vertebral bodies. Ankylosis of cervical facet joints and bridging syndesmophytes usually presented together if they appeared, but they were not always observed concurrently during the disease course.

It is generally accepted that the presence of facet joint ankylosis is associated with (bridging) syndesmophytes. In line with previous studies [5, 15, 16], our results revealed a high correlation between facet joint damage and vertebral body damage as assessed by individual radiographic scoring methods (i.e., the methods of de Vlam et al. and mSASSS, respectively). In addition to higher cervical mSASSS, higher levels of inflammatory markers, more severe sacroiliitis, and more frequent hip involvement were noted among patients who had ankylosed cervical facet joints; these characteristics might have led to more frequent use of TNF inhibitors in this patient group. More prevalent uveitis and less involvement of peripheral joints were exhibited by those with cervical facet joint ankylosis, as shown in previous reports [14, 16, 22]. Our observations with a higher rate of comorbid peripheral joint disease in the absence of cervical facet joint ankylosis are likely explained by the fact that the involvement of peripheral joints in AS is associated with less severe axial disease [8, 22].

Identifying that vertebral bodies (or facet joints) are more often affected or whether syndesmophyte formation precedes facet joint ankylosis will have implications for understanding the pathophysiology of AS [23]. Immunohistological analyses have demonstrated direct inflammatory factor involvement and subsequent ankylosis in the AS facet joint [24, 25]. However, there have been conflicting results among clinical studies with respect to the spinal structures in which ankylosis occurs preferentially. Some studies have suggested that facet joints are primarily involved in AS, and ankylosis of facet joints seems to precede bridging syndesmophytes because of a greater frequency of ankylosed facet joints without bridging syndesmophytes than bridging syndesmophytes without facet joint ankylosis [14, 26]. In contrast, other studies have offered more frequent observations of bridging syndesmophytes without ankylosed facet joints but rare observations of facet joint ankylosis without bridging syndesmophytes, suggesting that bridging syndesmophytes develop before ankylosis of the facet joints [9]. These seemingly contradictory results might be partially explained by variations in imaging modalities, scoring methods, or spinal segments examined, as well as heterogeneity of the study populations. However, studies addressing facet joint involvement have mostly been cross-sectional or conducted over a relatively short-term duration (~ 4 years). Given the within-individual variations of spinal disease progression in AS [27], longitudinal data collection accounting for dynamic processes of structural changes should be applied to facet joint assessment as they have been for vertebral bodies. Thus, we compared configurations between facet joint ankylosis without bridging syndesmophytes and bridging syndesmophytes without facet joint ankylosis within each follow-up period; we found similar proportions throughout the follow-up periods, ranging from 11.8% to 28.8% and 11.8% to 23.5%, respectively. Thus, spinal structural damage in AS can arise from any of the facet joints or vertebral bodies, with no preference for region or sequence.

Concerning radiographic involvement per vertebral level, facet joint ankylosis was most frequently observed at the C2–C3 level, consistent with previous reports [16]. However, studies using computed tomography exhibited a relatively even distribution of ankylosed facet joints throughout the cervical spine [11, 23]. Because some normal variants of C2–C3 facet joints can appear fused on lateral radiographs depending on the angle of oblique orientation [28], overestimation of ankylosis can occur. Conversely, ankylosis seemed to affect C6–C7 facet joints less frequently, likely due to inclusion of patients with advanced disease whose spinal radiographs were performed when they were in a stooped posture. In this position, the visibility of posterior spinal structures at the lower cervical vertebral level is likely to be compromised on lateral-view images; therefore, they were excluded from the analysis, although facet joints at the corresponding level might have been ankylosed. Further studies assessing the range of vertebral levels over which facet joints can be scored reliably would be meaningful.

Another matter for consideration is assessment of radiographic involvement of lumbar facet joints. Studies have reported difficulties in fine assessment of lumbar facet joints in standard lateral radiographs, probably because of their more prominent vertical orientation [29]. The method of de Vlam et al. for visualisation of lumbar facet joints is based on oblique-view radiographs; however, oblique-view lumbar images are often omitted [14]. Thus, radiographic abnormalities of lumbar facet joints were not assessed in this study. Along these lines, a newly proposed scoring method by Maas et al. [30] incorporates the cervical facet joint score (according to de Vlam et al.) into the total mSASSS to enhance visualisation of radiographic progression. This method is supported by our results showing equal effects of facet joint ankylosis and bridging syndesmophytes in the cervical spine.

This study has several limitations. We acknowledge limitations related to the facet joint scoring method, which has been validated less often than the mSASSS. There are currently fewer data available on the scoring method of de Vlam et al., and further validation of this approach is necessary in a long-term follow-up study of a large population. While radiographic scoring was performed with readers blinded to all clinical data while aware of the chronological order of the radiographs (the most sensitive method for detecting changes) [31], this may not be the best approach for scoring facet joint abnormalities. Because both the anterior and posterior structures of the spine are visible in a single radiographic image, the chronological approach might result in an overestimation of progression, especially in facet joints, due to expectation bias. Additionally, limitations inherent to the retrospective nature of the investigation include variable follow-up periods among individuals, making it difficult to determine the duration of ankylosis development in the facet joints. Instead, we stratified the follow-up period into 4-year increments by arbitrarily defining the threshold interval to detect radiographic progression. However, there are few studies on appropriate intervals between radiograph measurements to ensure sufficient sensitivity to capture structural changes in facet joints. Further studies are required to determine the minimum follow-up interval for achieving an acceptable degree of sensitivity to monitor changes.

## Conclusions

In conclusion, AS patients appear to have comparable structural damage in cervical facet joints and cervical vertebral bodies, as assessed by the presence of facet joint ankylosis and bridging syndesmophytes, respectively, on routine spinal radiographs. If visible, both cervical facet joints and syndesmophytes in the vertebral bodies should be assessed for ankylosis since facet joint ankylosis and bridging syndesmophytes usually present together, and patients with ankylosed cervical facet joints are more likely to have a greater disease burden.

## Electronic supplementary material

Below is the link to the electronic supplementary material.

Supplementary Material 1

## Data Availability

The datasets generated and/or analysed during the current study are not publicly available due to limitations of ethical approval involving the patient data and anonymity but are available from the corresponding author on reasonable request.

## Abbreviations

AS: Ankylosing spondylitis
BASDAI: Bath Ankylosing Spondylitis Disease Activity Index
BASRI-hip: Bath Ankylosing Spondylitis Radiology Hip Index
CI: Confidence interval
CRP: C-reactive protein
ESR: Erythrocyte sedimentation rate
HLA-B27: Human leucocyte antigen B27
ICC: Intraclass correlation coefficient
IQR: Interquartile range
mSASSS: Modified Stoke Ankylosing Spondylitis Spine Score
SD: Standard deviation
TNF: Tumour necrosis factor.
