# Supplementary material for: Radiographic involvement of cervical facet joints in ankylosing spondylitis: a longitudinal analysis in correlation with vertebral body lesions
Source: BMC Rheumatol. 2023 Jun 7;7:11. doi: 10.1186/s41927-023-00334-x (PMC10245667; doi:10.1186/s41927-023-00334-x)
Supplement: Supplementary file 1 — Supplementary Material 1 [file 41927_2023_334_MOESM1_ESM.docx]

**Supplementary Table 1** Correlations between cervical facet joint score and radiographic scores of individual skeletal regions or clinical parameters

|  | Cervical facet joint score (0–15) | |
| --- | --- | --- |
|  | r (correlation coefficient) | *P*-value |
| Age, years | 0.215 | < 0.001 |
| Symptom duration, years | 0.357 | < 0.001 |
| ESR, mm/h | 0.156 | < 0.001 |
| CRP, mg/dL | 0.064 | 0.09 |
| BASDAI (0–10) | 0.082 | 0.200 |
| Cervical mSASSS (0–36) | 0.717 | < 0.001 |
| Lumbar mSASSS (0–36) | 0.423 | < 0.001 |
| Total mSASSS (0–72) | 0.616 | < 0.001 |
| Sacroiliitis grade (0–4)^a^ | 0.379 | < 0.001 |
| BASRI-hip grade (0–4)^b^ | 0.372 | < 0.001 |

^a^Calculated by averaging the grades of the right and left sacroiliac joints according to the modified New York criteria

^b^Calculated by averaging the BASRI-hip grade of the right and left hip joints

BASDAI, Bath Ankylosing Spondylitis Disease Activity Index; BASRI-hip, Bath Ankylosing Spondylitis Radiology Hip Index; CRP, C-reactive protein; ESR, erythrocyte sedimentation rate; mSASSS, modified Stoke Ankylosing Spondylitis Spine Score
